# New Biopolymer Nanoparticles Improve the Solubility of Lipophilic Megestrol Acetate

**DOI:** 10.3390/molecules21020197

**Published:** 2016-02-06

**Authors:** Malwina Lachowicz, Michał Kołodziejczyk, Marek Lukosek, Jacek Kosno, Paulina Olszewska, Paweł Szymański

**Affiliations:** 1 Department of Applied Pharmacy, Medical University of Lodz, ul. Muszyńskiego 1, Lodz 90-151, Poland; malwina.lachowicz@umed.lodz.pl; 2 Surface-Active Agent Plant “ICSO Blachownia”, ul. Energetyków 9, Kędzierzyn-Koźle 47-225, Poland; lukosek.m@icso.com.pl (M.L.); kosno.j@icso.com.pl (J.K.); 3 Department of Pharmaceutical Chemistry, Drug Analysis and Radiopharmacy, Medical University of Lodz, ul. Muszyńskiego 1, Lodz 90-151, Poland; paulina.olszewska@umed.lodz.pl; 4 Laboratory of Radiopharmacy, Department of Pharmaceutical Chemistry, Drug Analysis and Radiopharmacy, Medical University of Lodz, ul. Muszyńskiego 1, Lodz 90-151, Poland; pawel.szymanski@umed.lodz.pl

**Keywords:** solubilization, surfactant, nanoparticles, megestrol acetate, Tween 80

## Abstract

As many substances are poorly soluble in water and thus possess decreased bioavailability, creating orally administered forms of these substances is a challenge. The objective of this study was to determine whether the solubility of megestrol acetate, a Biopharmaceutical Classification System (BCS) class II drug, can be improved by using a newly-synthesized surfactant (Rofam 70: a rapeseed methyl ester ethoxylate) and compare it with two references surfactants (Tween 80, Pluronic F68) at three different pH values. Spectrophotometry was used to compare the solubility profiles in the presence of three tested surfactants at pH 5.0, 7.4 and 9.0. Rapeseed methyl ester ethoxylate was found to improve the solubility of the BCS Class II drug and increase its bioavailability; It increased drug solubility more effectively than Pluronic F68. Its cytotoxicity results indicate its possible value as a surfactant in Medicine and Pharmacy.

## 1. Introduction

Oral intake is the most sought-after route of drug administration due to its convenience and ease of use by patients. However, 40% to 70% of newly-discovered chemical entities for use in pharmaceuticals are poorly soluble in water. This solubility problem is one of the main causes of difficulties with delivering existing drugs, and represents an obstacle for formulation scientists [1,2,3,4]. Low solubility leads to decreased bioavailability, incomplete release from formulation and limited possibilities in choosing delivery technologies. Hence, the discovery and implementation of innovative methods of drug formulation are regarded as high priorities for researchers [5].

A range of methods can be used to improve solubility, e.g., the formation of liposomes or surfactant micelles; however, some of these methods can have a harmful influence on organs, such as the liver or kidney [6]. Appropriate selection of surfactants contributes to improvements of drug solubility in water: the process being known as solubilization. Surfactants may act as solubilizers, and micellar solubilization increases the oral bioavailability of drugs with poor water solubility [7]. The bioavailability of drugs mainly depends on their solubility in gastrointestinal tract fluids [4] and dissolution is the rate-determining step for drug absorption [3]. The solubility and stability of the pharmaceutical product are both important considerations when designing new drugs based on new technology [8].

During solubilization, micelles are created. As these micelles have a size of less than 100 nm, they can be regarded as nanoparticles. In recent years, they have played important roles in Medicine and Pharmacy associated with improving the safety of drugs and enabling their direct delivery to the site of action [9]. In addition, nanoparticles are used to reduce the side effects and toxicity of drugs, to improve their pharmacokinetic properties and ensure their controlled and sustained release [10,11].

Megestrol acetate (MA), 17 α-acetyloxy-6-metylpregna-4,6-diene-3,20-dione has high permeability and poor water solubility, and thus is classified as a BCS class II drug [12], making it a strong candidate for this study. MA treats loss of appetite, reduces suffering in patients with endometrial or advanced breast cancer and is also used to eradicate undernourishment associated with AIDS [8,12,13,14,15]. The dissolution of BCS class II drugs is determined by the rate of drug absorption [3].

A new surfactant, rapeseed methyl ester ethoxylate, or Rofam 70, has recently been synthesized. Ethoxylation reactions were carried out in a pressure reactor with a capacity of 2 dm^3^, made of stainless steel and equipped with a mechanical stirrer, heating mantle, cooling coil, automatic dosing of ethylene oxide and also with measurement and control equipment controlled by computer. Hydrophobic substrate was mixed with a catalyst and fed into the reactor. After loading the reactor and checking the tightness of the raw material, charge was dried at 130 °C under an atmosphere of nitrogen for 30 min to remove traces of water. Then the reactor temperature was raised to the reaction temperature and started the automatic dosing of ethylene oxide. After the introduction of ethylene oxide, the reaction mixture was post-cured for a further 50 min at the reaction temperature, in order to increase the substrates to react. After cooling to about 60 °C the reactor was purged with nitrogen and the product was discharged, weighed and subjected. The aim of this study was to investigate the effect of Rofam 70 on the solubility of megestrol acetate (MA) at three different pH values, with Tween 80 and Pluronic F68 being used as references for comparison purposes. The results would give an insight into its suitability for medical and pharmaceutical applications.

## 2. Results and Discussion

### 2.1.Structural Analysis

The mass analysis of Rofam 70 (ethoxylated fatty acid methyl esters of rapeseed oil: average degree of ethoxylation, 70 moles TE/mol ester) revealed peaks at 44 Da intervals in the mass ranges 1100 to 2100 Da and 2100 to 4700 Da. This mass corresponds to an oxyethylene group (CH_2_CH_2_O).

**Figure 1:**
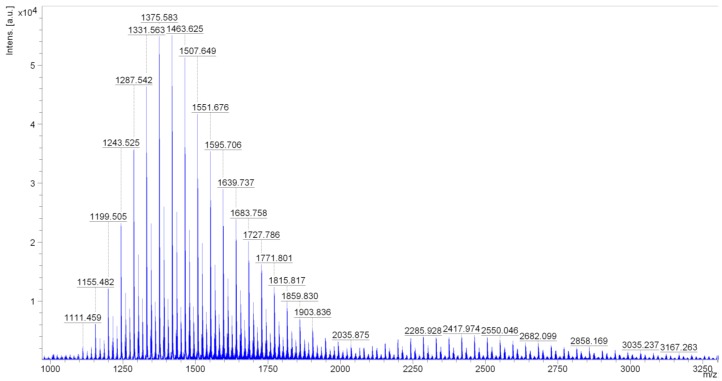
Matrix Assisted Laser Desorption Ionization (MALDI) mass spectrometry of the sample Rofam 70 in the range of 1000–3200 Da.

The main series of peaks in the mass range 1100 to 2100 Da corresponds to the structure of the ethoxylated fatty acid methyl ester R-COO(CH_2_CH_2_O)_n_CH_3_, where *n* = 18–40, and the main components of this fraction are at *n* = 22–29. The heights of these peaks are over half the height of the peak with the highest intensity (1419.6 *m*/*z*) (Figure 1).

However, the main series of peaks in the mass range 2000 to 4700 Da correspond to the structure of diesters of fatty acids and polyglycol R-COO(CH_2_CH_2_O)_n_OC-R, where *n* = 34–58 (including peaks with the intensity > 1000 a.u., to 3123.2 *m*/*z*). The main components of this fraction are at *n* = 35–50; the heights of these peaks are over half the height of the peak with the highest intensity (2462.0 *m*/*z*).

These results confirm that Rofam 70 consists of two fractions. The first being C_17_H_33_COO(CH_2_CH_2_O)_n_CH_3_, where *n* = 22–29, and the second being C_17_H_33_COO(CH_2_CH_2_O)_n_OCC_17_H_33_, where *n* = 35–50.

Tween 80 is the commercial name of polioxyethylene sorbitan monooleate [16].

### 2.2.GCP and HPLC Analysis

**Figure 2:**
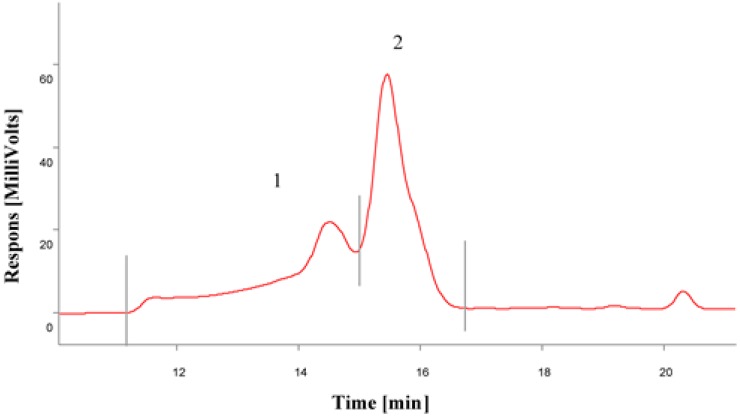
Gel permeation chromatography (GPC) gel chromatogram for fractions 1 (C_17_H_33_COO(CH_2_CH_2_O)_n_OCC_17_H_33_) and 2 (C_17_H_33_COO(CH_2_CH_2_O)_n_CH_3_).

**Figure 3:**
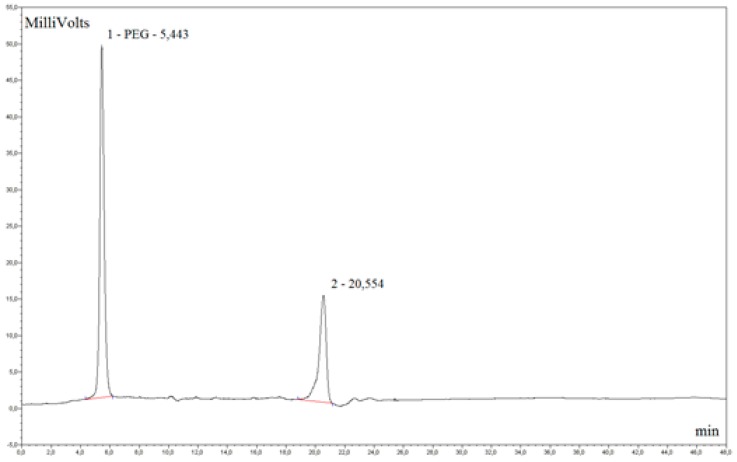
High performance chromatography (HPLC) chromatogram, where the higher peak represents fraction 2 (C_17_H_33_COO(CH_2_CH_2_O)_n_CH_3_) and the smaller peak represents fraction 1 ***(***C_17_H_33_COO(CH2CH2O)_n_OCC_17_H_33_).

The gel permeation chromatography (GPC) analysis of the oxyethylation products of rapeseed oil fatty acid methyl esters indicates the presence of two fractions. Fraction 1 consists of C_17_H_33_COO(CH_2_CH_2_O)_n_OCC_17_H_33_, diesters of fatty acids and polyglycols, and represents about 36% of total content of the oxyethylation products, while fraction 2 is C_17_H_33_COO(CH_2_CH_2_O)_n_CH_3_-oxyethylated fatty acid methyl esters, making up about 64% of the total content (Figure 2 and Figure 3).

### 2.3. Cytotoxicity Assay

**Figure 4:**
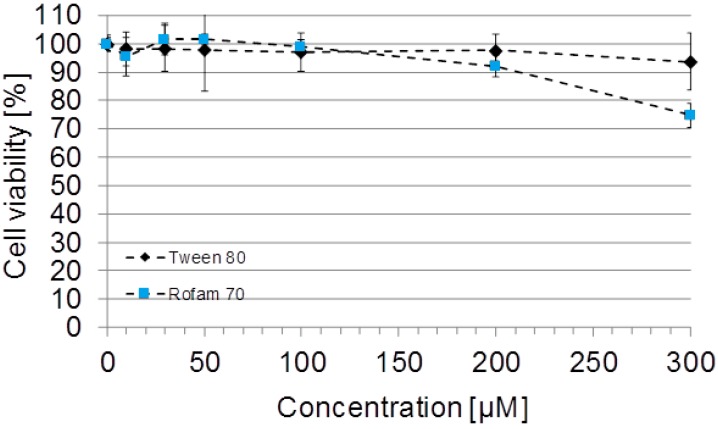
The effect of Rofam 70 and one of the tested positive controls, Tween 80 on A549 cell viability. Viable cells were quantified by water soluble tetrazolium salt (WST)-1 assay after 48 h of culture in the presence of the indicated compounds at concentrations ranging from 10 to 300 μM. Data is expressed as mean ± SD, *n* = 3.

The effect of Rofam 70 on cell viability was assessed using WST-1 assay. The cells were exposed to a wide concentration range of the tested compounds (10 µM to 300 µM) for 48 h. Tween 80, PEG 40 and Poloxamer 188, a commercial surfactant, were used as reference compounds. The results showed that Rofam 70 at concentration of 200 µM and lower did not exhibit cytotoxic effect on A549 cells (Figure 4). However, when cells were exposed to Rofam 70 at concentration of 300 µM we observed decreased number of viable cells to 74.8% ± 4.4% (Figure 4). Culture of A549 cells in the presence of reference compounds (Tween 80, PEG 40 and Poloxamer 188) at all tested concentrations (10–300 µM) had no significant effect on A549 cell viability. The results demonstrated that Rofam 70 does not affect cell viability when used up to 200 µM concentration.

### 2.4. Solubility

For all three tested surfactants, the solubilization of MA was more effective when centrifugation was not used. When comparing surfactants, maximum solubility was obtained in solutions containing Tween 80, with similar results being obtained for Rofam 70. Samples treated with Pluronic F68 gave poorer results (Table 1). These results indicate that Rofam 70 may be used as a surfactant. A similar compound was studied by Renkin *et al.* and by Smidrkal *et al.* in these papers while here was tested a new surfactant [17,18].

**Table 1:** Equilibrium solubility of megestrol acetate (mg/100 mL) in various pH/surfactant systems where NC—non-centrifuged, C—centrifuged.

Surfactant Concentration	Solubility (mg/100 mL) in Three Different pH		
	5.0	7.4	9.0
0.5% Tween 80	6.976 NC	7.873 NC	5.514 NC
	2.406 C	2.146 C	2.280 C
1% Tween 80	12.960 NC	11.359 NC	10.595 NC
	5.580 C	4.754 C	4.279 C
1% Pluronic F68	3.021 NC	2.621 NC	2.322 NC
	0.191 C	0.178 C	0.235 C
2% Pluronic F68	1.631 NC	2.098 NC	1.894 NC
	0.102 C	0.206 C	0.184 C
1% Rofam 70	6.696 NC	5.228 NC	6.233 NC
	2.526 C	2.773 C	2.288 C
2% Rofam 70	10.385 NC	7.541 NC	7.352 NC
	5.187 C	4.906 C	4.749 C

Substances which are poorly soluble in water are characterized by decreased bioavailability, and their pharmaceutical development presents a challenge [19]. One such substance is MA, 17-(acetyloxy)-6-methyl-pregna-4,6-diene-3,20-dione, a drug which is used to treat loss of appetite and weight in patients with AIDS, as well as endometriosis and advanced breast cancer. Due to its insolubility in water and high permeability, MA is classified as BCS class 2 [20]. As more than 40% of newly discovered chemical entities are insoluble in water, it is necessary to identify more effective formulations before they can be implemented [21].

Micelles are created during solubilization, and due to their small size, they can be classified as nanoparticles. These offer many advantages, and these are demonstrated by Rofam 70. Nanotechnology, which has recently grown into a pharmaceutical science in its own right, is an innovative area whose implementation offers the creation of delivery systems that not only improve solubility but also increase the safety and efficacy of drugs. These implementations can also contribute to reducing the dosing frequency of the drug, which can solve the problems associated with non-adherence to regular medication administration regimes [22]. Our findings indicate that Rofam 70 is suitable for use as a surfactant, and together with those obtained for the other two surfactants, they provide much encouragement for further studies on such compounds.

## 3. Materials and Methods

### 3.1. Structure Analysis

#### 3.1.1. Mass Spectroscopy

The registration of the mass spectra was performed using a Bruker autoflex speed Matrix Assisted Laser Desorption Ionization-Time of flight (MALDI-TOF) mass spectrometer. A Sartorius MC5 microbalance was used to weigh the sample, templates and salts applied in these studies.

The sample was spotted on a MALDI plate by a “dried droplet method”. The analyzed sample is dissolved in a volatile solvent. The sample solution is then mixed with excess solution template and if necessary, small quantities of substances facilitating ion formation (e.g., Trifluoroacetic acid salts) are added. Around 1 µL of the mixture is placed on a stainless steel plate and the solvent is left to evaporate in an air stream.

The following procedure was used to prepare the sample. About 5 mg of the sample was dissolved in 1 mL of tetrahydrofuran (THF-T. Baker) and left to dissolve. As the sample did not completely dissolve, 100 µL distilled water was added until completely dissolved.

The templates used were 2,5-dihydroxybenzoic acid (2,5-DHB), 1,8,9-trihydroxyanthrone (dithranol), 3-indoleacrylic acid (IAA) and anthracene. The 2,5-DHB was obtained from Bruker, and the other templates from Sigma-Aldrich. The template used in tetrahydrofuran solution with a concentration of 10 g/L. Used salts: sodium trifluoroacetate (NaTFA). The salts used in the tetrahydrofuran solution with a concentration of 0.1 mol/L.

Application to Maldi plate:

- proportion of temple solutions:sample:salt = 25 µL:5 µL:0/1 µL; 25 µL:10 µL:0/1 µL; 10 µL:10 µL:0 µL; 5 µL:10 µL:0 µL; 5 µL:20 µL:0 µL
- applied 1 µL of mixture.

The used parameters:

Mode of operation of the spectrometer: reflectron.

Ionization: positive.

Spectra were registered in the mass range to 5500 Da.

The best spectra obtained for the solution of 3-indoleacrylic acid (IAA):solution of sample = 10 µL:10 µL.

#### 3.1.2. GPC and HPLC Analysis

Molecular weight distribution was analyzed by gel permeation chromatography (GPC) on a reverse phase column at a critical point of adsorption and/or normal phase. Polystyrene columns were used and tetrahydrofuran were applied as solvent and mobile phase.

High performance chromatography (HPLC) was used to separate polyglycol homologues and determine the total concentration of analytes in samples. A reversed phase analytical column (ODS 250 × 2.1 mm) was used. The mobile phase was water–acetonitrile (35:65, *v*:*v*). Analytes were detected with an evaporative light scattering detector (ELSD), which is an ideal instrument for detecting compounds with no UV chromophores. The eluent stream passes through the column, where the solvent is evaporated and a mist of tiny sample particles is left. These scatter a light beam, and the extent of the light scattering is proportional to the amount of sample present.

### 3.2. Cytotoxicity

#### 3.2.1. Cell Culture

A549 cells were purchased from the European Collection of Cell Cultures (ECACC, Salisburg, UK). The cells were grown in Dulbcco’s Modified Eagle’s Medium (DMEM) (Euroclone, Pero (MI), Italy) supplemented with 10% heat-inactivated fetal bovine serum, FBS (Biochrome, Berlin, Germany) and antibiotic (100 units/mL penicillin and 100 µg/mL streptomycin) (Euroclone). Incubation took place in a humidified atmosphere with 5% CO_2_ at 37 °C.

#### 3.2.2. Cytotoxicity Assay

The concentration of tested compounds causing 50% inhibition of cell growth (IC_50_) was determined using a colorimetric WST-1 assay (Millipore, Billerica, MA, USA) following the manufacturer’s instructions. Mitochondrial dehydrogenase of viable cells converted water soluble tetrazolium salt (WST-1) to a yellow formazan product, with the amount of formazan dye formed directly correlating to the number of live cells. A549 cells were seeded in a 96-well plate at a density of 5000 cells per well. After 24 h, the cells were exposed to vehicle control and tested compounds at various concentrations from 10 to 300 µM. Stock solutions of tested compounds were prepared in DMSO and diluted in complete medium to obtain final concentrations. After 48 h of incubation with the tested compounds, the cells were treated with the WST-1 reagent and the incubation was continued for another 3 h. Absorbance was determined at 440 nm using a microplate reader (Synergy H1, Bio-Tek). Cell viability was expressed as a percentage of the control values. The IC_50_ values were calculated from a concentration–response curve using Microsoft Excel. Data was presented as the mean ± SD of three independent experiments performed in triplicate.

### 3.3. Solubility Determination

#### 3.3.1. Method I

The procedure was carried out using mixtures of 0.2 M KH_2_PO_4_/0.2 M NaOH at different proportions to form buffer solutions with pH values of 5.0, 7.4 or 9.0. To these mixtures was added either Tween 80 to make a final concentration of 0.5% or 1%, Pluronic F68 to a final concentration of 1% or 2%, or Rofam 70 to a final concentration of 1% or 2%. In addition, control solutions were made using the same buffer solutions at the same pH values but without surfactants. Into the vials containing 40 mL of this media was added an excess amount of MA powder (Sigma-Aldrich, St. Louis, MO, USA); purity is ≥99% determined by HPLC (20 mg). Then the vials were shaken at 120 c.p.m. for 4 h at 30 °C (Elpin type 357 shaker with water bath, Elpin plus, Lubawa, Poland) and the samples were centrifuged at 3000 rpm for 15 min (Hettich Zentrifugen Micro 22R Centrifuge). Drug concentration was registered spectrophotometrically (Nicolet evolution 300) in the range of 220–320 nm as the absorption maximum of MA is approximately 287 nm. All measurements were performed in triplicate [23].

#### 3.3.2. Method II

The second method is similar to the first, but omits the centrifugation step. Briefly, solutions of buffers 0.2 M KH_2_PO_4_/0.2 M NaOH were prepared at pH values of 5.0, 7.4 and 9.0, either with surfactant (Rofam 70, Tween 80, Pluronic F68) at concentrations shown in Table 1, as detailed in Method I above. As with Method I, control solutions were made without surfactants. To the prepared solutions was added an excessive amount of MA. The vials containing these solutions were shaken at 120 c.p.m. for 4 h at 30 °C (Elpin type 357 shaker with a water bath). After this time, the samples were measured spectrophotometrically in the range of 220–320 nm using a Nicolet Evolution 300 spectrophotometer: the absorption maximum of megestrol acetate being 287 nm. All measurements were performed in triplicate [23].

## 4. Conclusions

The present study was performed to determine whether a newly-synthesized compound, rapeseed methyl ester ethoxylate (Rofam 70), could be used as a surfactant. Our findings indicate that it has comparable cytotoxicity to the commonly-used compounds Tween, PEG40 and Poloxamer. In addition, the impact of Rofam 70 on the solubility profile of lipophilic drugs belonging to BCS class 2 was compared with those of other surfactants (Tween 80, Pluronic F68) at three different pH values. The obtained results suggest that Rofam 70 may be used to replace previously used surfactants such as Pluronic in medication. Studies have shown that ethoxylated fatty acid methyl esters are very biodegradable and non-toxic and also they are referred to as one of the surfactants of future [17,24]. Due to availability and production scale these substances are considered attractive costs raw material for the production of surfactants. The proposed method of synthesis ethoxylated fatty acid methyl esters is one-stage and non-waste in contrast to synthesis of fatty acid esters and ethoxylated sorbitol. Therefore, it can be assumed that ethoxylated fatty acid methyl esters may be more economical substitutes and alternatives to commonly uses surfactants which are general called Tween.

Many widely-used medications may benefit from the use of more appropriate surfactants such as Rofam 70 in their formulation, resulting in their greater solubility and hence, more efficient delivery, and future research should reflect this.
